# Progress in the Diagnosis and Classification of Pituitary Adenomas

**DOI:** 10.3389/fendo.2015.00097

**Published:** 2015-06-12

**Authors:** Luis V. Syro, Fabio Rotondo, Alex Ramirez, Antonio Di Ieva, Murat Aydin Sav, Lina M. Restrepo, Carlos A. Serna, Kalman Kovacs

**Affiliations:** ^1^Department of Neurosurgery, Hospital Pablo Tobon Uribe and Clinica Medellin, Medellin, Colombia; ^2^Laboratory Medicine, Division of Pathology, St. Michael’s Hospital, University of Toronto, Toronto, ON, Canada; ^3^Department of Endocrinology, Universidad Pontificia Bolivariana, Medellin, Colombia; ^4^Department of Neurosurgery, Australian School of Advanced Medicine, Macquarie University, Sydney, NSW, Australia; ^5^Nisantasi Pathology Group, Istanbul, Turkey; ^6^Division of Endocrinology, Clinica Medellin, Medellin, Colombia; ^7^Laboratorio de Patologia y Citologia Rodrigo Restrepo, Department of Pathology, Clinica Medellin, Medellin, Colombia

**Keywords:** diagnosis, genetics, pathology, acromegaly, multiple endocrine neoplasia type 1, pituitary adenoma, familial isolated, Carney complex

## Abstract

Pituitary adenomas are common neoplasms. Their classification is based upon size, invasion of adjacent structures, sporadic or familial cases, biochemical activity, clinical manifestations, morphological characteristics, response to treatment and recurrence. Although they are considered benign tumors, some of them are difficult to treat due to their tendency to recur despite standardized treatment. Functional tumors present other challenges for normalizing their biochemical activity. Novel approaches for early diagnosis, as well as different perspectives on classification, may help to identify subgroups of patients with similar characteristics, creating opportunities to match each patient with the best personalized treatment option. In this paper, we present the progress in the diagnosis and classification of different subgroups of patients with pituitary tumors that may be managed with specific considerations according to their tumor subtype.

## Introduction

Pituitary adenomas are benign tumors representing approximately 15–20% of intracranial neoplasms (1). They can present with pituitary dysfunction, neurological deficits (especially visual impairment), and/or invasion into parasellar compartment and/or sphenoid sinuses. Endocrinologically active tumors present different challenges to normalize their hormonal production (2). Initially considered as sporadic tumors, some of them are associated with familial syndromes (3). Morphologically, pituitary adenomas represent a heterogeneous group of tumors and their meticulous pathological classification is required (4).

Early diagnosis of pituitary tumors is advisable and their proper classification is of paramount importance for treatment and prognostic purposes (5). Pituitary adenomas have been classified according to the clinical, radiological, and endocrinological findings, tumor size, and invasiveness. In morphologic/pathologic studies, they were initially classified on the basis of their tinctorial characteristics with hematoxylin–eosin stain as acidophilic, basophilic, amphophilic, and chromophobic adenomas. Immunohistochemical investigation achieved a major progress identifying hormone production by tumor cells. Electron microscopy added further information by defining the cell type of the tumor and its ultrastructure. Recent molecular/genetic/epigenetic methods are still in their initial phase and more research is required.

Despite the standard protocols of treatment, some pituitary adenomas may have a clinically aggressive course with tendency to recur, becoming giant in size, and/or invading surrounding structures (6). At present, there is no consensus regarding the diagnosis of aggressive adenomas. The early recognition of aggressiveness and the prediction of pituitary tumor behavior remain a challenge (7). There are no specific and universally accepted biomarkers yet. The correct diagnosis of histological subtypes of pituitary adenomas may predict clinical aggressive behavior in some cases. Here, we present a novel progress in the diagnosis of acromegaly and different subgroups of pituitary adenomas which, in the opinion of the authors, deserve special mention due to their characteristic clinical behavior and special considerations in terms of diagnosis, treatment and follow-up.

## Progress in Diagnosis

### Early diagnosis in acromegaly

Despite the characteristic physical manifestations and significant comorbidities, the diagnosis of acromegaly may take some years. Its slow and insidious course and its changes are frequently unnoticed by the patient, family members, friends, and physicians. Although new surgical and medical treatments have emerged along with new developments in laboratory tests, the diagnosis of patients with acromegaly has not changed in many years. Reid et al. (8) studied 324 patients from the same institution during two different periods of time and no significant differences were found in terms of clinical symptoms, size of the tumor, and delay at diagnosis in both groups. The clinical recognition of acromegaly has not improved over the last 25 years. Novel approaches for an early diagnosis of acromegaly have been proposed. Based on photographs, Miller et al. (9) assessed the accuracy of diagnosis of acromegaly between a computer program and general physicians. The diagnostic accuracy of the computer model was 86%, whereas that of the physicians was only 26%. In a similar study, Schneider et al. (10) compared classification accuracy of acromegaly by means of a face analysis software. Their program correctly classified 71.9% of patients versus 63.2 and 42.1% by experts and general internists, respectively. In both studies, computer analysis reached diagnosis better than physicians. By using photographs and special software, it can be possible to detect and diagnose acromegalic changes early, even in cases with low clinical suspicion. This innovative approach opens the possibility that, in the near future, using specialized software, mobile apps, and capillary networks, diagnosis can be made earlier. In a society consisting of more well-informed patients, astonishing technological advances and easy internet access, novel ways of improving early diagnosis of acromegaly should be implemented.

## Progress in Classification

### Familial pituitary tumors

**Figure 1:**
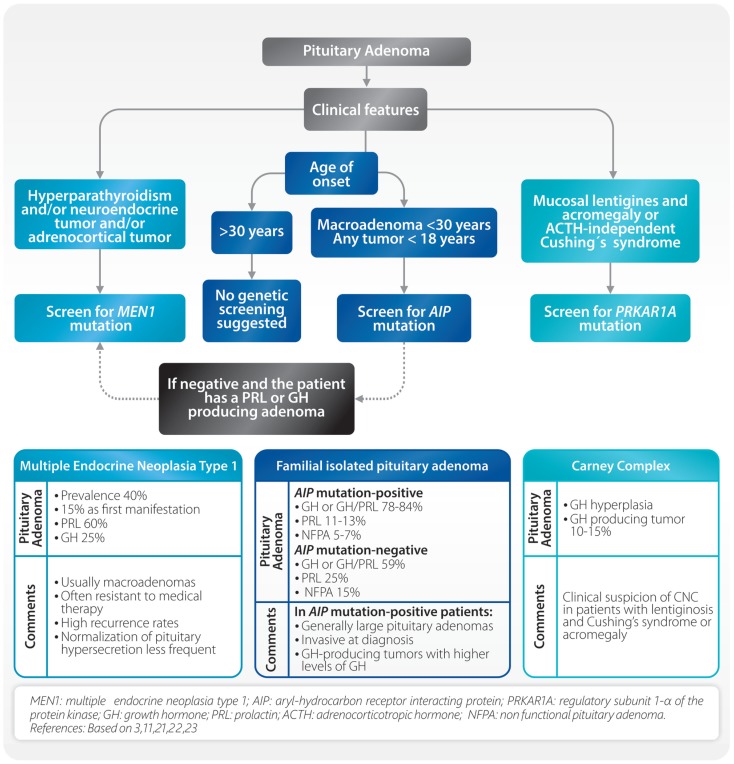
**Considerations in apparently sporadic pituitary adenomas and common familial syndromes**.

Recent findings have revealed the existence of familial pituitary tumor syndromes (3, 11). These syndromes represent a group of diseases with different genetic background and variable phenotype. The most frequently seen are multiple endocrine neoplasia type 1 (MEN1), familial isolated pituitary adenoma (FIPA), and Carney complex (CNC) (Figure 1). Other uncommon familial syndromes include somatotropinoma/paraganglioma (12), pituitary blastoma (13), and X-linked acrogigantism (X-LAG) syndrome (14, 15). These will not be discussed since they are not in the scope of this review.

Multiple endocrine neoplasia type 1 (MEN1) is an autosomal-dominant disorder characterized by tumors of the pituitary, parathyroid, endocrine gastrointestinal tract, endocrine pancreas, and adrenal cortex (16). Localized on chromosome 11q13, *MEN1* is a tumor suppressor gene encoding menin, a nuclear protein involved in transcriptional regulation, genome stability, cell division, and proliferation. About 5–10% of patients with MEN1 phenotype may not harbor mutations in the coding region of the *MEN1* gene. In a small group of patients, mutations in *CDKN1B* were found. This infrequent MEN1-like syndrome was named MEN4 (17). Patients with MEN1 syndrome usually have a family history of MEN1 and *MEN1* gene mutations, which can be identified in 70–95% of patients (16). Clinical diagnosis is established when two of these features are present or when one feature is present together with a first-degree relative with established MEN1. Pituitary tumors in MEN1 patients are present from 10 to 60% and they can be the first clinical manifestation in up to 15% of the cases. Pituitary adenomas associated with MEN1 differ from sporadic ones (18). In MEN1, they are usually diagnosed at earlier ages, frequently macroadenomas, often resistant to medical therapy and with high recurrence rates (18). Although PRL and GH-producing adenomas are the most frequent, almost all types of pituitary adenomas can occur (19). Due to the fact that primary hyperparathyroidism is present in the majority (approximately 90%) of MEN1 patients, parathyroid hyperplasia/adenoma/carcinoma should be ruled out in all cases of apparently sporadic pituitary adenomas. In this setting, examination of the parathyroid gland and its function can help to detect possible MEN1 patients (Figure 1). Characterization of the *MEN1* gene product, menin, can also be achieved using immunohistochemistry (IHC) by site-specific MEN1 antibodies. Here, menin can be localized to the nucleus of pituitary adenomas removed from patients with MEN1 (20).

Familial isolated pituitary adenoma is characterized by the presence of pituitary adenomas in two or more members of a family. Prevalence of FIPA is not known but it is probably similar to MEN1 (21). In 20% of these families, a mutation on the aryl-hydrocarbon receptor interacting protein (*AIP*) gene is found. In patients with apparently sporadic pituitary adenomas under the age of 18 years, *AIP* mutations are present in approximately 20% (11). FIPA shows an autosomal dominant pattern with incomplete penetrance and a wide variation between families. The affected families can be classified as *AIP* mutation-positive or *AIP* mutation-negative, and according to the phenotype as: (a) homogenous, if the same type of pituitary adenoma is present, or (b) heterogeneous, if different types of tumors occur within the same family. There are some differences between *AIP* mutation-positive and *AIP* mutation-negative patients (Figure 1). In *AIP* mutation-positive patients, the mean age of onset is lower (20–24 years), they are predominantly males (63.5%), and they present in 85% of the cases with GH or GH/PRL producing tumors. *AIP* mutation-positive acromegalic patients have larger and more invasive tumors (22) (Figure 1). At present, published data are inconclusive in regards to the clinical behavior of *AIP* mutation-negative patients. Diagnosis of AIP-related FIPA relies on the detailed analysis of the pituitary adenoma based on hormone secretion, imaging, and histologic findings. Using IHC techniques, in cases where the patient is *AIP* mutation-positive, immunostaining for aryl hydrocarbon receptor nuclear translocator (ARNT) and aryl hydrocarbon receptor (AHR) will demonstrate a decrease of ARNT protein expression and an increase of AHR localization in the nucleus (23, 24). Increased expression of nuclear AHR in pituitary adenomas with *AIP* mutations may indicate a loss of signal in phosphodiesterase 2 enzyme (PDE2A), which normally regulates cyclic adenosine monophosphate (cAMP). As a result, there is an increase in cAMP concentration which may lead to the subsequent formation of a pituitary adenoma (25).

Carney complex, an autosomal dominant syndrome, is characterized by the following features: multiple skin lesions (blue nevus, spotty skin pigmentation, and mucosal lentigines), cardiac myxoma, acromegaly, psammomatous melanocytic schwannoma, thyroid carcinoma, multiple hypoechoic thyroid nodules, and primary pigmented nodular adrenocortical disease (PPNAD) with Cushing’s syndrome (26). It is produced by an inactivating mutation of the regulatory subunit 1-α of the protein kinase A (PRKAR1A). The diagnosis can be established when two of the above mentioned major features occur, or in the presence of one major feature and an inactivating *PRKAR1A* mutation, or a first-degree relative with CNC. Clinical manifestations are variable even within members of the same family and one-third of the patients present as simplex (sporadic) cases. Lentiginosis is the most common feature of CNC (70%) consisting of small, 2–10 mm brown to black macule on the lips, eyelids, ears, and genitals, and can be present at birth and acquire their clinical characteristics at puberty (26). ACTH independent Cushing’s syndrome due to PPNAD is the main endocrine manifestation (60%) followed by acromegaly due to pituitary adenoma or adenohypophyseal hyperplasia.

In all cases of apparently sporadic pituitary adenomas, an effort for recognizing a familial syndrome must be made because in many instances the clinical behavior and response to treatment are different (18). Primary hyperparathyroidism must be ruled out, because this is present in 90% of MEN1 patients. If other clinical features of MEN1 are present, *MEN1* mutation analysis should be performed. In patients with tumors diagnosed before 18 years of age, as well as patients with macroadenomas before 30 years of age, it is advisable to screen *AIP* and/or *MEN1* mutations (27). Clinical suspicion of CNC must arise in patients with lentiginosis and Cushing’s syndrome or acromegaly (Figure 1) (28).

## Progress in Morphological Classification

### Somatotroph pituitary adenomas

GH-producing adenomas constitute 10–15% of all pituitary adenomas. The more common morphological subtypes are densely and sparsely granulated somatotroph adenomas (5, 29). Densely granulated somatotroph adenomas (DGSA) are acidophilic tumors with diffuse and intense GH immunopositivity. Immunostaining demonstrates low molecular weight keratin (LMWK), Cam5.2, in a diffuse perinuclear pattern. Sparsely granulated somatotroph adenomas (SGSA) are composed of chromophobic or mildly acidophilic cells. Immunopositivity for GH is variable, it is usually scarce and of low intensity. The fibrous bodies, initially described by electron microscopy and corresponding to accumulation of paranuclear cytokeratins, are the characteristic feature of this tumor type. With IHC, using LMWK (Cam5.2) the identification of paranuclear “dot” pattern is characteristic of this tumor type and correlates with the electron microscopic finding (30). In recent studies, a correlation between these subtypes and clinical response was confirmed (31, 32). Clinical, histopathological, and radiological characteristics of acromegalic patients were subjected to cluster analysis by Cuevas–Ramos et al. (31). They classified acromegalic patients in three groups based on significant differences in morphology, tumor aggressiveness, treatment responsiveness, expression profile of somatotroph surface receptors, markers of cell senescence, and disease outcomes. Type-1 comprised patients with densely granulated GH tumors, micro or macroadenomas with higher expression of somatostatin receptor 2 (SSTR2), and p21, a cyclin-dependent kinase inhibitor (CDKI) (33, 34). Type-2 comprised patients with mixed densely and sparsely granulated GH tumors and Type-3 comprised patients with sparsely granulated GH tumors. They found that Type-3 adenomas were aggressive macroadenomas, and comprised patients with adverse outcomes, in spite of receiving more treatment modalities. This proposed classification may identify distinctive patterns of disease aggressiveness and outcomes in acromegalic patients and it can be a useful tool for selection criteria in clinical studies. Definite clinical distinction is useful between DGSA and SGSA, because their recognition is helpful as far as prognosis and treatment is concerned (5, 35, 36). Assessment of somatostatin receptors 2 and 5 (SSTR2, SSTR5) stains can be useful to predict the response to medical treatment (37–39).

### Crooke’s cell adenomas

ACTH-secreting adenomas constitute 10–15% of all pituitary adenomas and present three different morphological subtypes. The densely granulated corticotroph subtype is the most common. This tumor is composed of basophilic or amphophilic, PAS positive cells, strongly immunopositive for ACTH and LMWK (Cam5.2) in a perinuclear pattern. By electron microscopy, numerous secretory granules are noted and bundles of keratin filaments are apparent around the nucleus, consistent with perinuclear LMWK (Cam5.2) immunostaining. Patients with Cushing’s disease and Nelson’s syndrome usually present this histologic subtype. Sparsely granulated corticotroph adenoma, the second subtype is rare. It is immunopositive for LMWK (Cam5.2) and variable for ACTH.

Crooke’s cell adenomas are the third subtype (40). In 1935, Crooke was the first to describe the histologic features of the adenohypophysis in patients with Cushing’s syndrome. He noticed that, in the presence of ACTH secreting adenomas, non-neoplastic corticotrophs often showed accumulation of intracytoplasmic, perinuclear hyaline material. These *Crooke’s cells* are considered corticotrophs which, in presence of glucocorticoid excess, undergo massive accumulation of perinuclear cytokeratin. Using LMWK (Cam5.2) immunostaining, a strong, ring-like pattern around the nucleus is seen, with displacement of ACTH immunoreactivity under the cell membrane. In some ACTH producing adenomas, for unknown reasons, there is a massive hyaline change in the majority of the cells, the same as the Crooke’s cells seen in the adenohypophysis of patients with glucocorticoid excess (40). The reasons why these cells produce ACTH, and at the same time display Crooke’s hyaline changes are not well understood. Tumors that contain so-called Crooke’s hyaline material in their cytoplasm of more than 50% of the cells are classified as Crooke’s cell adenomas (40, 41). As mentioned, Crooke’s cell tumors may produce ACTH causing Cushing’s disease in 75% of the cases, or may be endocrinologically silent. They frequently exhibit aggressive clinical behavior, with high recurrence rate and invasiveness (41); thus, strict surveillance and eventual multimodal treatment are recommended.

### Aggressive pituitary adenomas

According to the World Health Organization (WHO), pituitary tumors are classified as typical adenomas, atypical adenomas, and carcinomas (42). The majority are typical adenomas, with monotonous histological features. They are slow growing, well demarcated, non-invasive adenomas, showing no major cellular and nuclear pleomorphism, few mitotic figures, and a Ki-67 nuclear index <3%. Atypical adenomas are tumors that disclose “atypical morphological features suggestive of aggressive behavior” (42), such as invasive growth, elevated mitotic index, a Ki-67 labeling index >3%, and extensive nuclear staining for the p53 protein. Pituitary carcinomas can only be diagnosed if cerebrospinal and/or systemic metastases are documented. They may develop by transformation from adenomas or arise *de novo* from non-tumorous adenohypophyseal cells. Pituitary carcinomas produce more often PRL or ACTH (43). However, GH, TSH, FSH, LH, or alpha subunit can also be produced or they may be immunohistochemically negative.

The WHO classification of typical and atypical adenomas does not correlate with clinical behavior. Neither all typical adenomas have a benign clinical evolution nor do all atypical adenomas have the tendency to recur or invade surrounding structures. Some tumors exhibit high rate of recurrence, resistance to conventional treatments, and invasion to the parasellar compartment and/or sphenoid sinuses, often requiring multiple surgeries. They seem to represent a distinct entity and may be defined as aggressive pituitary adenomas (6, 44, 45). Their distinction and definition are controversial and elusive. Morphologically, they are pleomorphic, contain mitotic figures, and have a rapid cell proliferative rate. The assessment of the Ki-67 nuclear labeling, using the MIB-1 antibody, can be the most useful tool. If the Ki-67 nuclear labeling index is more than 10%, the tumor could be classified as aggressive adenoma, although there is no agreement on this (7, 43). A recent clinicopathological classification, which takes into account proliferation markers, invasion to cavernous and sphenoid sinus and size, has been proposed (46). For the criteria of invasiveness, the authors considered histological and/or radiological signs of cavernous sinus or sphenoid sinus invasion. For the assessment of cell proliferation, at least two of the three following criteria had to be present: Ki67 ≥3%, 2 or more mitoses per 10 high-power fields, and p53 immunopositivity. Pituitary adenomas were classified in five groups according to the invasion, presence of proliferation, and metastasis. After an 8-year follow-up, invasive and proliferative tumors had an increased probability of tumor persistence or progression. Furthermore, based on the fact that six out of the eight carcinomas in their series were classified as invasive and proliferative at the first surgery, the authors postulated that they are possibly malignant tumors without metastasis and proposed that the term *tumor suspected of malignancy* be used for them (47). Other authors have suggested the term *in situ* carcinoma or a premetastatic pituitary carcinoma in the sellar phase (43, 48).

Aggressive adenomas can produce GH, PRL, ACTH, TSH, FSH, LH, alpha subunit, or they can be immunonegative. Some aggressive adenomas are silent, not over-producing hormones, and unassociated clinically with hormonal excess. The question arises whether aggressive pituitary adenomas have malignant potential. It may well be that some of them are actually carcinomas without any accompanying solid evidence of metastases. Some carcinomas may have their malignant nature already before any metastases can be detected and may metastasize afterward. These are important questions; their resolution is of crucial significance. Obviously, new biomarkers have to be investigated to resolve the biologic behavior of aggressive pituitary adenomas and carcinomas. There may be two alternative possibilities that carcinomas may develop: either *de novo* from normal non-tumorous adenohypophyseal cells or transform gradually from adenoma to aggressive adenoma and carcinoma (1, 49). Further studies are needed to conclusively recognize and identify aggressive pituitary tumors.

### Pituitary transcription factors

**Figure 2:**
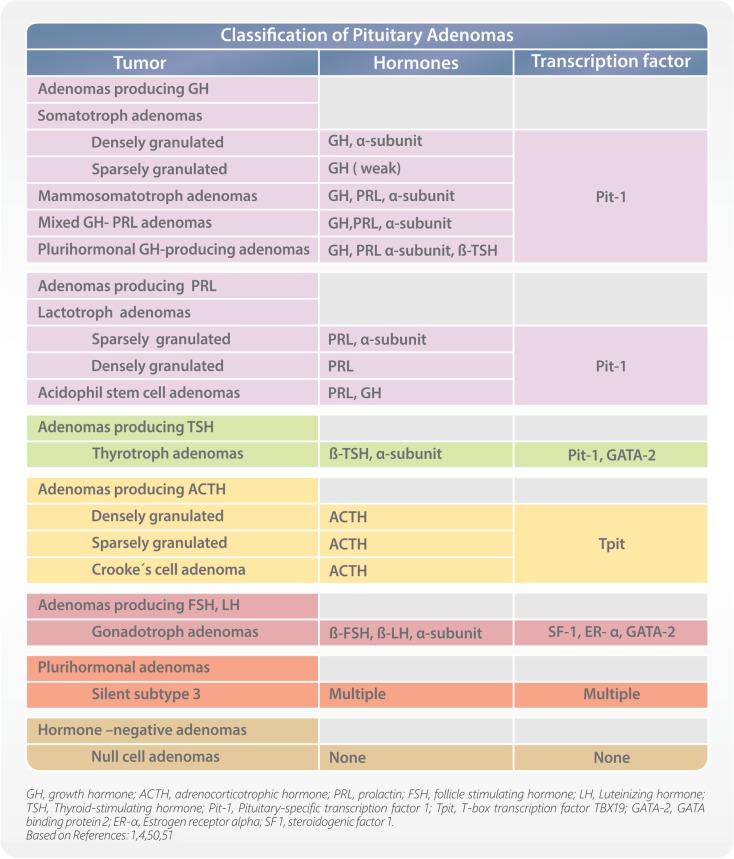
**Classification of pituitary adenomas**.

During development, the process of cell differentiation is coordinated by specific transcription factors. They also have some estimated roles in determining the cytodifferentiation and hormone production of pituitary adenomas, and can help in their classification. Pituitary tumors are monoclonal benign adenomas, and secrete specific hormones reflective of their differentiated cell of origin. Pituitary-specific transcription factor 1 (Pit-1) defines cells that can produce GH, PRL, and/or TSH. T-box transcription factor TBX19 (Tpit) identifies corticotrophs. Estrogen receptor alpha (ER-α) cooperates with Pit-1 to enhance PRL secretion; therefore, coexpression of Pit-1 and ER-α is seen in lactotrophs. Guanine-Adenine-Thymine-Adenine binding protein 2 (GATA-2) appears to be an important contributor to thyrotroph development and is coexpressed with Pit-1 in thyrotrophs. Expression of steroidogenic factor 1 (SF1), ER-α, and GATA-2 identify gonadotrophs. Immunohistochemical demonstration of transcription factors helps to accurately classify pituitary adenomas (Figure 2), especially in cases of low or absent of identifiable hormone content (4, 50).

The discovery of adenohypophyseal cell plasticity has changed our thinking in the classification of pituitary tumors (51). Earlier, it was universally accepted that pituitary cells can produce only one hormone, cannot change their morphology and hormonal production, and cannot transform to another cell type. This concept has been proven to be inaccurate and the findings of adenohypophyseal cell plasticity changed our understanding completely. At present, conclusive evidence indicates that adenohypophyseal cells can transform to another cell type, they can produce more than one pituitary hormone, and can change their morphology and phenotype. Adenohypophyseal cell plasticity also alters our views in regards to pituitary tumors. The factors that affect pituitary cell transformation are not well known and more work is needed to confirm the mechanism behind cell plasticity. Adenohypophyseal cell plasticity can have a major influence in the proper classification of pituitary tumors. It is possible that pituitary adenoma cells can change their morphology and phenotype and transform to another cell type, known as transdifferentiation. Moreover, some reports have also showed that benign, slow-growing adenomas can transform to aggressive adenomas or carcinomas (52).

### Markers of transformation of pituitary adenomas

The search for new, reliable markers to predict the behavior of pituitary tumors is ongoing (1). Some markers have shown promise; however, their utility to conclusively indicate pituitary tumor transformation is debatable. Early studies of markers such as galectin-3 and cyclooxygenase II (Cox-2) have shown inconsistent results. Others, such as matrix metalloproteinase’s (MMPs), p27, p21, vascular endothelial growth factor (VEGF), CD34, hypoxia-inducible factor 1 alpha and pituitary tumor transforming gene (PTTG), show promising roles as markers for indicating tumor behavior but more work is needed to elucidate their role and prognostic value.

## Conclusion

Proper classification of pituitary tumors is an important area of research. Novel approaches for early diagnosis as well as different perspectives on their classification may help to identify subgroups of patients that share similar characteristics (31). The recognition of these subgroups creates opportunities to match each patient with the best personalized treatment option. In the subgroups of patients discussed here, some specific characteristics may predict their clinical behavior and their response to treatment. New techniques may help us to reach a more accurate classification and personalized and precise treatment options for patients harboring pituitary adenomas (53).

## Conflict of Interest Statement

The authors declare that the research was conducted in the absence of any commercial or financial relationships that could be construed as a potential conflict of interest.
